# Supplementary material for: Use of Antiplatelet Agents and Survival of Tuberculosis Patients: A Population-Based Cohort Study
Source: J Clin Med. 2019 Jun 27;8(7):923. doi: 10.3390/jcm8070923 (PMC6678265; doi:10.3390/jcm8070923)
Supplement: Supplementary file 1 [file jcm-08-00923-s001.pdf]

Table S1. Agent category and included drugs.

**Category**	**Drugs included**
Irreversible cyclooxygenase inhibitors	aspirin
Adenosine diphosphate (ADP) receptor inhibitors	clopidogrel, ticagrelor, ticlopidine
Phosphodiesterase inhibitors	cilostazol
Glycoprotein IIB/IIIA inhibitors	abciximab, eptifibatide, tirofiban
Adenosine reuptake inhibitors	dipyridamole

Table S2. Definition of comorbidities and other variables

**Comorbidity**	**Definition**
Diabetes mellitus	At least one inpatient or three outpatient compatible diagnoses (ICD-9-CM code 250) and prescription of insulin or oral hypoglycemic agents for ≥90 defined daily dose (DDD) within 1 year
Chronic obstructive pulmonary disease	At least two outpatient or inpatient records with compatible diagnoses (ICD-9-CM codes 491~492, 496) and prescription of at least two COPD-specific medications or one COPD-specific medication plus at least one airway medication during a period of 90 days
Cancer	Compatible ICD-9-CM code (140~208) from the Registry for Catastrophic Illness Patient Database (RCIPD), which is a separate file section of the National Health Insurance (NHI) database
End-stage renal disease	ICD-9-CM code 585 in the RCIPD
Acquired immunodeficiency disease	Two or more records with compatible diagnoses (ICD-9-CM code 042, V08) and prescription of highly active antiretroviral therapy during a period of 180 days
Pneumoconiosis	ICD-9-CM codes 500~505 in the RCIPD
Bronchiectasis	At least two outpatient records or one inpatient record of ICD-9-CM code 494 during a period of 90 days
Rheumatoid arthritis	At least three outpatient records of ICD-9-CM code 714 by a rheumatologist or dermatologist or one inpatient record of ICD-9-CM code 714 during a period of 180 days

Ankylosing spondylitis	At least three outpatient records of ICD-9-CM code 720 by a rheumatologist or dermatologist or one inpatient record of ICD-9-CM code 720 during a period of 180 days
Psoriasis	At least three outpatient records of ICD-9-CM code 696 by a rheumatologist or dermatologist or one inpatient record of ICD-9-CM code 696 during a period of 180 days
Coronary artery disease	At least two outpatient records of ICD-9-CM codes 410~414 or receiving either coronary angioplasty or coronary bypass surgery (procedure codes: 33076B, 33077B, 33078B, 68023B, 68024B, 68025B, N26002, N26003)
Ischemic stroke	At least two outpatient or one inpatient records of ICD-9-CM code 433.01, 433.11, 433.21, 433.31, 433.81, 433.91, 434.01, 434.11, 434.91 within 1 year
Hypertension	At least three outpatient records or one inpatient record of ICD-9-CM code 401-404 during a period of 180 days
Transplant	ICD-9-CM code V42 or 996 in RCIPD
Steroid use	use of steroids with cumulative dose more than 90 DDD within 180 days prior to TB diagnosis

Table S3. ICD-9-CM and ICD-10-CM codes* used to define intracranial hemorrhage and gastrointestinal bleeding

Category	ICD-9-CM codes	ICD-10-CM codes
Intracranial hemorrhage	430, 431, 432	I60, I61, I62
Gastrointestinal bleeding	456.0, 456.20, 530.21, 530.7, 530.82, 531.0, 531.2, 531.4, 531.6, 532.0, 532.2, 532.4, 532.6, 533.0, 533.2, 533.4, 533.6, 534.0, 534.2, 534.4, 534.6, 535.01, 535.11, 535.21, 535.31, 535.41, 535.51, 535.61, 535.71, 537.83, 537.84, 569.85, 569.86, 562.02, 562.03, 562.12, 562.13, 569.3, 578, 568.81	K92.0, K92.1, I85.0, I98.20, I98.3, K22.10, K22.12, K22.14, K22.16, K25.0, K25.2, K25.4, K25.6, K26.0, K26.2, K26.4, K26.6, K27.0, K27.2, K27.4, K27.6, K28.0, K28.2, K28.4, K28.6, K29.0, K63.8, K31.80, K31.88, K55.8, K91.8, I84.1, I84.4, I84.8, K55.20, K62.5, K92.2

* Starting from 2016, ICD-10-CM codes were used in the NHI database to replace ICD-9-CM codes

Table S4. Defined daily doses (DDDs) of drugs in tuberculosis patients with and those without antiplatelet use

	Before PS matching						After PS matching					
	Overall	Antiplatelet users (*n* = 9497)			Non-user	*p* value*	Overall	Antiplatelet users (*n* = 8864)			Non-user	*p* value*
	(N = 74,753)	All (*n* = 9497)	ASA (*n* = 7764)	Non-ASA (*n* = 1855)	(*n* = 65,256)		(*n* = 17,728)	All (*n* = 8864)	ASA (*n* = 7281)	Non-ASA (*n* = 1704)	(*n* = 8864)	
**DDD of drug**												
irCOXi	20.8 ± 18.9	147.3 ± 77.9	177.0 ± 49.6	38.8 ± 69.8	2.4 ± 10.3	< 0.001	77.8 ± 47.3	149.2 ± 78.0	178.5 ± 49.6	39.6 ± 70.7	6.4 ±16.5	< 0.001
ADPRi	5.0 ± 13.8	33.7 ± 66.7	9.1 ± 34.1	156.4 ± 56.8	0.8 ± 6.2	< 0.001	18.0 ± 39.2	33.3 ± 66.8	9.0 ± 34.1	157.5 ± 57.5	2.8 ±11.7	< 0.001
PDEi	0.1 ± 3.2	0.1 ± 3.2	0.1 ± 1.8	0.6 ± 7.1	0.01 ± 0.65	< 0.001	0.1 ± 1.6	0.1 ± 2.7	0.04 ± 1.48	0.5 ± 6.0	0.04 ± 1.47	0.052
GIIB/IIIAi	0.03 ± 0.98	0.01 ± 0.13	0.0 ± 0.13	0.01 ± 0.16	0.00 ± 0.04	< 0.001	0.00 ± 0.08	0.00 ± 0.11	0.00 ± 0.10	0.01 ± 0.16	0.00 ± 0.05	0.001
ARi	1.2 ± 6.5	3.8 ± 14.2	3.3 ± 12.2	6.7 ± 22.7	0.9 ± 5.4	< 0.001	5.3 ± 18.6	3.9 ± 14.2	3.3 ± 12.4	6.78 ± 22.9	2.3 ± 9.0	< 0.001
Statins	7.7 ± 29.6	27.6 ± 59.8	25.9 ± 57.5	40.5 ± 73.1	4.8 ± 25.2	< 0.001	19.5 ± 48.8	28.2 ± 60.6	26.5 ± 58.3	41.4 ± 74.2	10.7 ± 37.0	< 0.001
NSAIDs	31.5 ± 59.9	38.6 ± 68.4	39.9 ± 69.8	33.2 ± 61.1	30.5 ± 58.7	< 0.001	39.5 ± 69.5	39.4 ± 69.3	40.7 ± 70.8	33.9 ± 61.5	39.6 ± 69.6	0.846
Metformin	9.0 ± 31.7	18.8 ± 46.3	20.1 ± 48.2	13.1 ± 36.8	7.6 ± 29.5	< 0.001	17.3 ± 44.3	19.4 ± 47.2	20.7 ± 49.1	13.5 ± 37.7	15.3 ± 41.4	< 0.001
Corticosteroids	29.8 ± 95.2	38.9 ± 115.7	36.5 ± 116.6	48.3 ± 108.8	28.5 ± 92.2	< 0.001	37.9 ± 110.4	36.4 ± 113.1	34.1 ± 114.3	46.1 ± 105.7	39.3 ± 107.6	0.077

Abbreviations: ADPRi, adenosine diphosphate receptor inhibitor; ARi, adenosine reuptake inhibitor; ASA, aspirin; GIIB/IIIAi, glycoprotein IIB/IIIA inhibitor; irCOOXi, irreversible cyclooxygenase inhibitor; NSAID, nonsteroidal anti-inflammatory drug; PAR-1i, protease-activated receptor-1 antagonist; PDEi, phosphodiesterase inhibitor. * Compared between all antiplatelet users and non-users.

Table S5. Defined daily doses (DDDs) of drugs in aspirin users (ASA), non-aspirin antiplatelet users (non-ASA), and non-antiplatelet users (non-users) after propensity-score matching

	**ASA (*n* = 7281)**	**Matched non-user (*n* = 7281)**	***p* value**	**Non-ASA (*n* = 1704)**	**Matched non-user (*n* = 1704)**	***p* value**
**DDD of drug**						
irCOXi	178.5 ± 49.6	6.2 ±16.2	< 0.001	39.6 ± 70.7	6.9 ± 17.0	< 0.001
ADPRi	9.0 ± 34.1	2.6 ± 11.3	< 0.001	157.5 ± 57.5	3.6 ± 13.5	< 0.001
PDEi	0.04 ± 1.48	0.04 ± 1.38	0.840	0.5 ± 6.0	0.1 ± 2.3	0.012
GIIB/IIIAi	0.00 ± 0.10	0.00 ± 0.05	0.043	0.01 ± 0.16	0.00 ± 0.05	0.018
ARi	3.3 ± 12.4	2.2 ± 8.7	< 0.001	6.8 ± 22.9	2.5 ± 9.5	< 0.001
Statin	26.5 ± 58.3	10.8 ± 37.2	< 0.001	41.4 ± 74.2	10.1 ± 34.3	< 0.001
NSAIDs	40.7 ± 70.8	40.3 ± 70.4	0.735	33.9 ± 61.5	36.4 ± 65.0	0.257
Metformin	20.7 ± 49.1	15.8 ± 42.3	< 0.001	13.5 ± 37.7	12.9 ± 36.6	0.646
Corticosteroids	34.1 ± 114.3	38.1 ± 105.6	0.028	46.1 ± 105.7	43.7 ± 111.7	0.526

Abbreviations: ADPRi, adenosine diphosphate receptor inhibitor; ARi, adenosine reuptake inhibitor; ASA, aspirin; GIIB/IIIAi, glycoprotein IIB/IIIA inhibitor; irCOXi, irreversible cyclooxygenase inhibitor; NSAID, non-steroid anti-inflammatory drug; PAR-1i, protease-activated receptor-1 antagonist; PDEi, phosphodiesterase inhibitor.
